# Sex differences of inflammatory and immune response in pups of Wistar rats with SIRS

**DOI:** 10.1038/s41598-020-72537-y

**Published:** 2020-09-28

**Authors:** Anna M. Kosyreva, Dzhuliia Sh. Dzhalilova, Olga V. Makarova, Ivan S. Tsvetkov, Natalia A. Zolotova, Marina A. Diatroptova, Elena A. Ponomarenko, Vladimir A. Mkhitarov, Dmitriy N. Khochanskiy, Liliya P. Mikhailova

**Affiliations:** 1 Department of Immunomorphology of Inflammation, Research Institute of Human Morphology, Tsyurupi str 3, 117418 Moscow, Russia; 2 Medical Institute of Peoples’ Friendship, University of Russia (RUDN University), Moscow, Russia

**Keywords:** Thymus, Bacterial infection, Acute inflammation, Sepsis

## Abstract

It is a common fact, that the content of sex hormones in humans and animals varies in different age periods. The functional state of the immune system also changes with age. However, sex differences studies of inflammatory and immune responses during puberty prevail in literature. Investigation of immune responses to LPS peculiarities in prepubertal females and males may contribute to the development of more effective immunotherapy and minimize side effects of children vaccination. Therefore, the aim of this work was to investigate the LPS-induced SIRS sex differences in prepubertal Wistar rats. Despite the absence of sex differences in estradiol and testosterone levels, LPS-induced inflammatory changes in liver and lungs are more pronounced among males. Males demonstrate the increasing neopterin, corticosterone levels and alanine aminotransferase (ALT) and aspartate aminotransferase (AST) activity. Not less important is that in females, demonstrating less morphological changes in liver and lungs, endotoxin level is tenfold higher, and corticosterone level decreases. Thus, endotoxin cannot be used as a marker of the severity of multiple organ failure in prepubertal period. The LPS-induced immune reactions in females and males are similar and are characterized by immunosuppression. Both females and males have decreased production of cytokines (IL-2, IL-4, TNF-α, TGF-β) and the absolute number of CD3 + and CD3 + CD8 + lymphocytes in blood. The acute atrophy of thymus and apoptosis of thymic cells are revealed in animals of both sexes. However, the number of CD3 + CD4 + T-helpers and CD4 + CD25 + Foxp3 + T-cells decreases only in females with SIRS, and in males there was a decrease of CD45R + B-cells. The least expressed sex differences in immune responses in the prepubertal period can be determined by the low levels of sex steroids and the absence of their immunomodulatory effect. Further studies require the identification of mechanisms, determining the sex differences in the inflammatory and immune responses in prepubertal animals.

## Introduction

Systemic inflammatory response syndrome (SIRS) is a severe state triggered by infectious agents (viruses, bacteria, fungi) and extensive tissue damage. According to numerous studies in puberty, the severity and mortality from infectious and inflammatory diseases, including SIRS and sepsis, are determined by sex^1–4^. Immune reactions to pathogens depend on the karyotype and sex steroids^5,6^. Females are usually considered to have more active immune system with higher rate of autoimmune diseases, more pronounced response to vaccination, and some extent of protection from severe infection diseases like sepsis^5,7–9^. In our previous work we revealed that LPS induced in adult males more pronounced inflammatory response than in females, but females demonstrated more effective cellular and humoral immunity reactions during SIRS^10^.

In comparison to adults, which are characterized by strongly detected differences in sex hormones concentration, the prepubertal period individuals demonstrate low levels of estradiol and testosterone and they do not differ between the sexes^11^. However, it was shown that the severity of acute and chronic inflammatory diseases varied in boys and girls of prepubertal age^6,12^. L. Bindl et al.^13^ showed that among children 1–12 months of age sepsis occurs in boys 2.8 times more often than in girls. During the age from 1 to 8 years, the frequency of sepsis decreases in boys, and the rates do not differ from those in 8 year-old girls.

Apparently, sex differences in the severity and mortality of infectious and inflammatory diseases in prepubertal children are determined not by sex hormones, but mostly by the karyotype. Some proteins of immune responses, including members of NF-kB signaling pathway, are encoded on the X chromosome^6,14^. Polymorphisms of X-linked genes and cellular mosaicism in females obviously could be a reason of sex differences in inflammatory and immune reactions that determine the course and outcome of the infectious diseases in prepubertal individuals. Nonetheless, A.K. Ghuman et al.^15^ didn’t reveal gender differences of severity and sepsis mortality rates in prepubertal individuals.

In order to find out whether there are differences in the immune and inflammatory response to SIRS in the prepubertal period we investigated morphological changes in the target organs to LPS, cytokines production and shift of lymphocytes subpopulations in pups of Wistar rats.

## Results

### Sex differences of hormonal changes in pups with SIRS

No differences in steroid sex hormone level were detected among female and male rats of the control groups. However, the concentration of corticosterone was higher in females than in males (Table 1).

**Table 1:** Concentration of hormones in the blood serum of female and male pups of Wistar rat 1 day after LPS injection.

Hormones	Groups	Sex		P^f-m^
		Female	Male	
Estradiol, pg/ml	Control^1^ (n = 8)	87.2 (75.6–93.4)	79.3 (70.4–89.9)	0.8
	LPS^2^ (n = 10)	81.0 (65.6–114.8)	110.6 (88.0–126.1)	0.3
	P^1–2^	0.8	**0.01**	
Free testosterone, pg/ml	Control^1^ (n = 8)	0.46 (0.26–0.79)	0.24 (0.19–0.39)	0.5
	LPS^2^ (n = 10)	0.43 (0.22–0.71)	0.49 (0.30–0.70)	0.6
	P^1–2^	0.8	0.1	
Total testosterone, ng/ml	Control^1^ (n = 8)	0.80 (0.44–1.02)	0.42 (0.33–0.65)	0.7
	LPS^2^ (n = 10)	0.70 (0.41–1.11)	0.84 (0.60–1.10)	0.5
	P^1–2^	0.9	**0.02**	
Corticosterone, ng/ml	Control^1^ (n = 8)	623.8 (494.5–692.5)	97.2 (57.9–214.7)	**0.0002**
	LPS^2^ (n = 10)	231.2 (103.1–279.6)	244.8 (181.2–462.5)	0.2
	P^1–2^	**0.0005**	**0.02**	

Notes: p^1–2^—statistical significance of differences between control and SIRS groups of the same sex; p^f-m^—statistical significance of differences between female and male of the same experimental group.

Bold - statistically significant difference, *p* < 0.05.

After LPS injection the concentration of estradiol and total testosterone increased only in males, when in females it was not detected. The content of corticosterone increased in males and decreased in females, so there were no sex differences between male and female corticosterone level with SIRS. The concentration of free testosterone in both females and males did not change after LPS injection.

### Sex differences of inflammatory response in pups with SIRS

#### Inflammatory changes in the lungs in female and male pups of Wistar rats with SIRS

**Figure 1:**
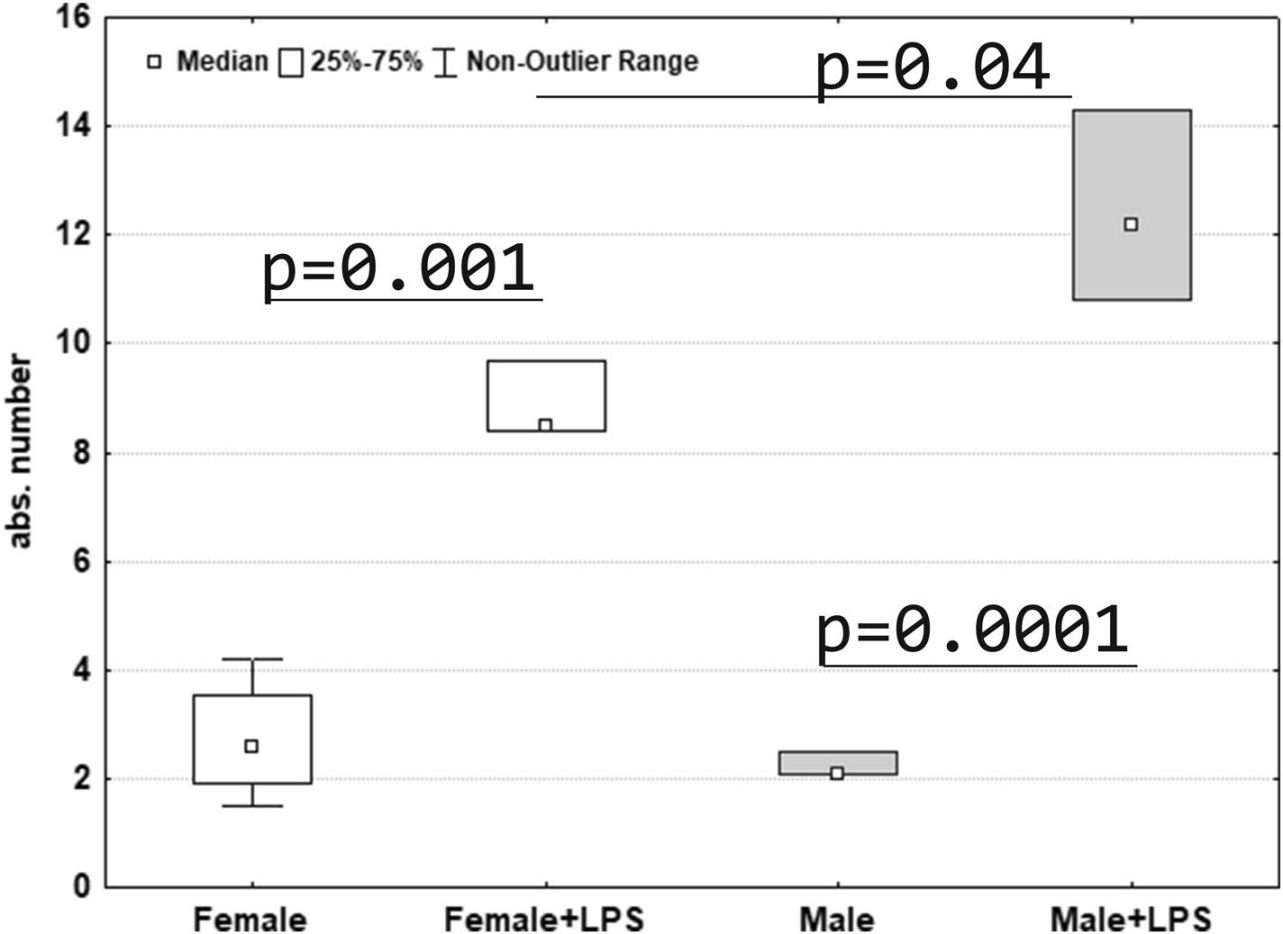
Absolute number of neutrophils in the intraalveolar septa of the lung of female and male pups of Wistar rats 1 day after LPS injection.

A day after the LPS injection local intraalveolar edema in the lung developed in 100% of females (in 10 pups out of 10) and in 80% of males (in 8 pups out of 10). Number of neutrophils in interalveolar septa significantly increased both in females and males. However, in males the number of neutrophils was 1.5 times higher than in females (Fig. 1).

#### Pathological changes in the liver in female and male pups of Wistar rats with SIRS

**Figure 2:**
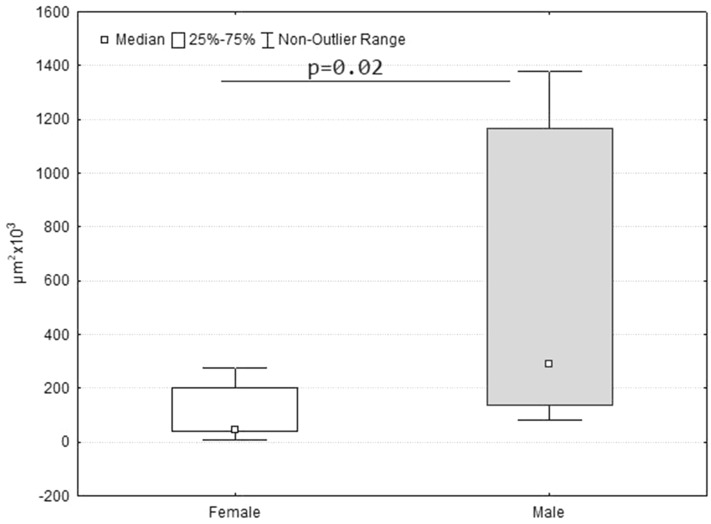
Area of necrosis (μm^2^ × 10^3^) in the liver of female and male pups of Wistar rat 1 day after LPS injection.

After LPS injection there were pathologic changes in the liver of both females and males: focal and widespread necrosis, vacuolar degeneration in hepatocytes of various severity (from less pronounced to expressed), hyperemia, stasis and sludge of sinusoid capillaries. However, males had larger area of necrosis in the liver than females (Fig. 2).

**Figure 3:**
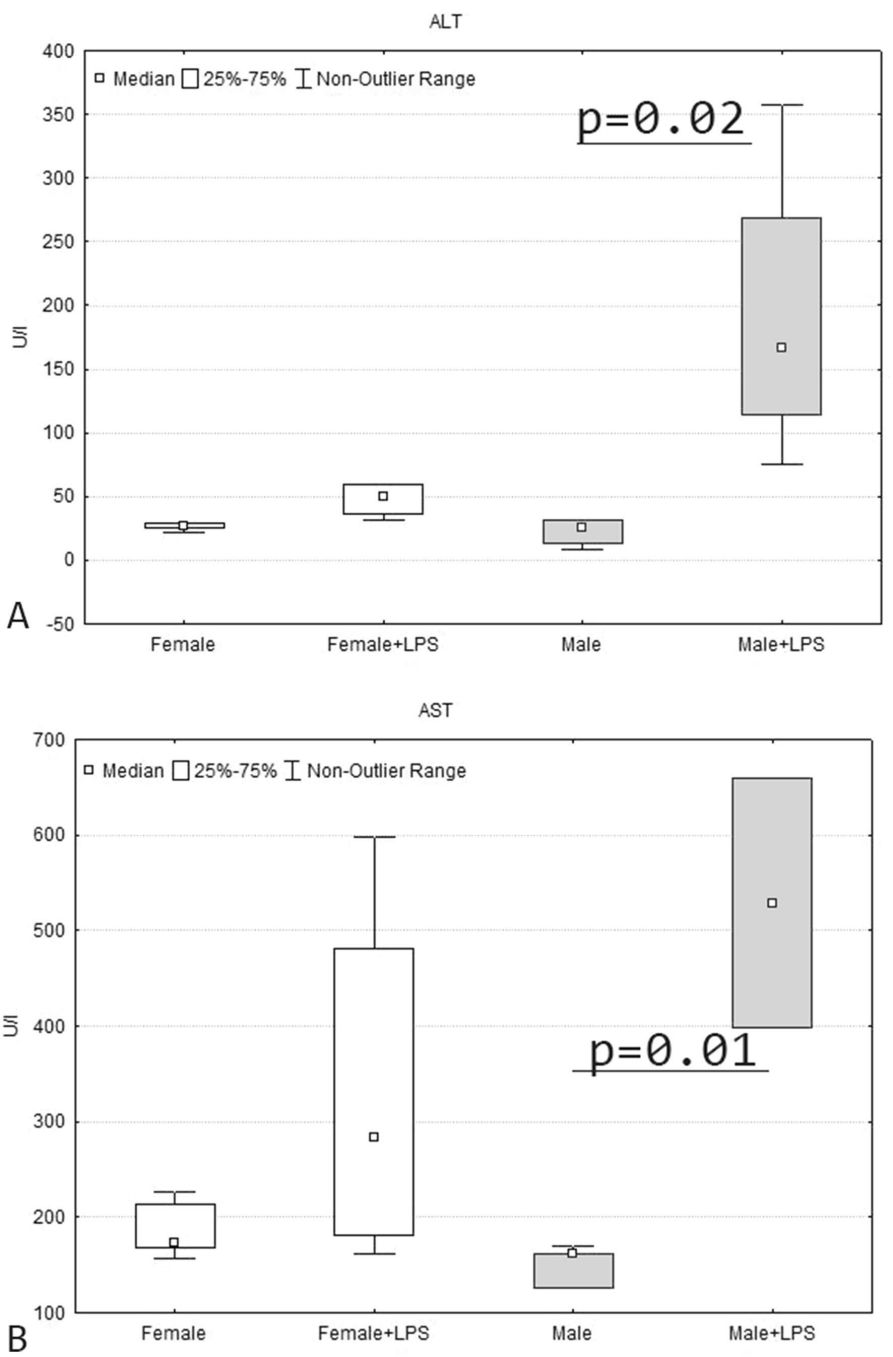
ALT (**A**) and AST (**B**) activity level in the serum blood of female and male pups of Wistar rat 1 day after LPS injection.

Activity level of ALT and AST increased in the serum only in males, whereas in females didn’t change (Fig. 3).

#### Changes of endotoxin level and neopterin, TGFβ, and C-reactive protein (CRP) concentration in the serum of female and male pups of Wistar rats with SIRS

After LPS injection the level of endotoxin both in females and males increased, but it was 10 times higher in females than in males (Table 2).

**Table 2:** Level of endotoxin, concentration of CRP, neopterin and TGFβ in the blood serum of female and male pups of Wistar rat 1 day after LPS injection.

	Groups	Sex		P^f-m^
		Female	Male	
Endotoxin, EU/ml	Control^1^ (n = 8)	2.6 (1.3–4.8)	2.3 (0.7–3.8)	0.6
	LPS^2^ (n = 10)	430 (417.5–451.3)	42.3 (9.6–188.8)	**0.03**
	P^1–2^	**0.00008**	**0.03**	
CRP, ng/ml	Control^1^ (n = 8)	3036 (2917–4239)	2507 (2249–2765)	0.06
	LPS^2^ (n = 10)	4594 (3207–4673)	3107 (3037–3646)	0.1
	P^1–2^	**0.03**	**0.04**	
Neopterin, nmol/l	Control^1^ (n = 8)	2.8 (2.4–4.6)	2.1 (1.9–2.3)	**0.01**
	LPS^2^ (n = 10)	4.6 (3.7–5.8)	3.2 (2.0–4.0)	**0.02**
	P^1–2^	0.05	**0.02**	
TGFβ, ng/ml	Control^1^ (n = 8)	41.3 (31.1–52.1)	57.3 (45.0–63,49)	0.1
	LPS^2^ (n = 10)	18.0 (13.8–26.6)	20.9 (17.4–31.3)	0.3
	P^1–2^	**0.01**	**0.001**	

Bold - statistically significant difference, *p* < 0.05.

The concentration of C-reactive protein (CRP) in pups of both sexes also increased after LPS injection and did not differ between male and female rats (Table 2).

The neopterin concentration in the serum of female pups of control group was higher than in males (Table 2). After LPS injection both in males and females, the concentration of neopterin increased, but it also was higher in females as compare with males.

The level of TGFβ in the serum of rats of both sexes decreased and did not had any sex differences after LPS injection (Table 2).

Thus, inflammatory reactions in pups with SIRS had the same features in rats of both sexes. They had elevation of CRP in blood serum, intraalveolar edema of the lung, vacuolar degeneration of hepatocytes, endotoxemia. However, there were sex differences: the mortality rate, severity of the pathological changes in the lung and liver, activity level of ALT and AST in the blood serum were higher in males than in females, but females had higher level of endotoxin in the blood.

### Sex differences of immune response after LPS injection

#### Morphological changes in the thymus of female and male pups of Wistar rats with SIRS

**Figure 4:**
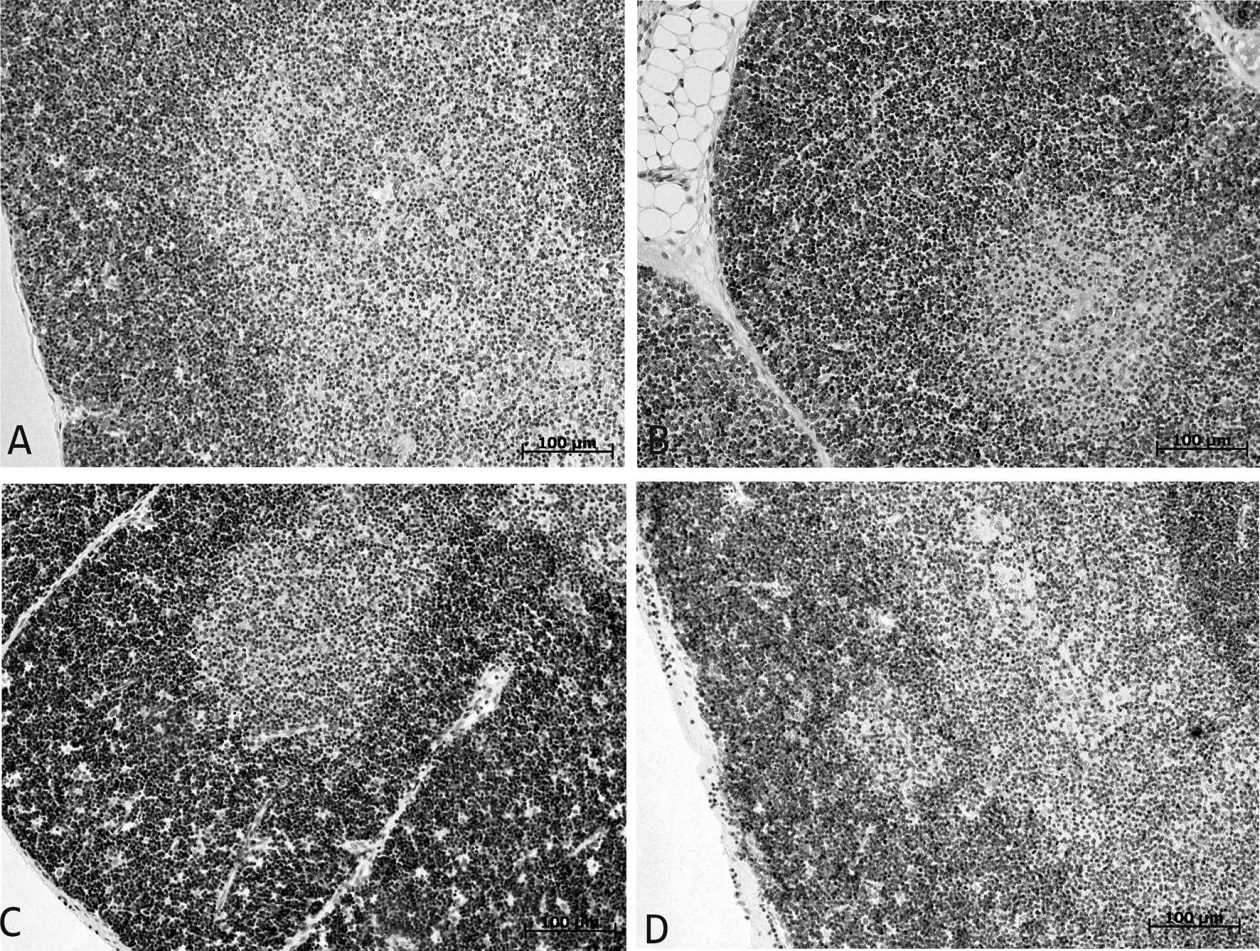
Morphological changes in the thymus of female (**A**,**C**) and male (**B**,**D**) pups of Wistar rats of control group (**A**,**B**) and 1 day after LPS injection (**C**,**D**). After LPS injection the ‘holes’ in the cortex are seen, these ‘holes’ had formed at the place of dying lymphocytes.

One day after the administration of LPS to female and male pups Wistar rats, signs of a mild atrophy of the thymus were found (Fig. 4). As in females, in males with SIRS the width of the thymic cortex was narrowed compared with their control groups (Table 3).

**Table 3:** Width of the cortex in the thymus of female and male pups of Wistar rat 1 day after LPS injection.

Groups	Sex		P^f-m^
	Female	Male	
Control^1^ (n = 8)	74.4 (68.5–75.6)	81.6 (80.7–86.2)	0.5
LPS^2^ (n = 10)	62.4 (44.8–70.0)	76.8 (71.4–80.3)	0.6
P^1–2^	**0.006**	**0.03**	

Bold - statistically significant difference, *p* < 0.05.

#### The relative number of Annexin + apoptotically dying cells in the thymus of female and male pups of Wistar rats with SIRS

**Figure 5:**
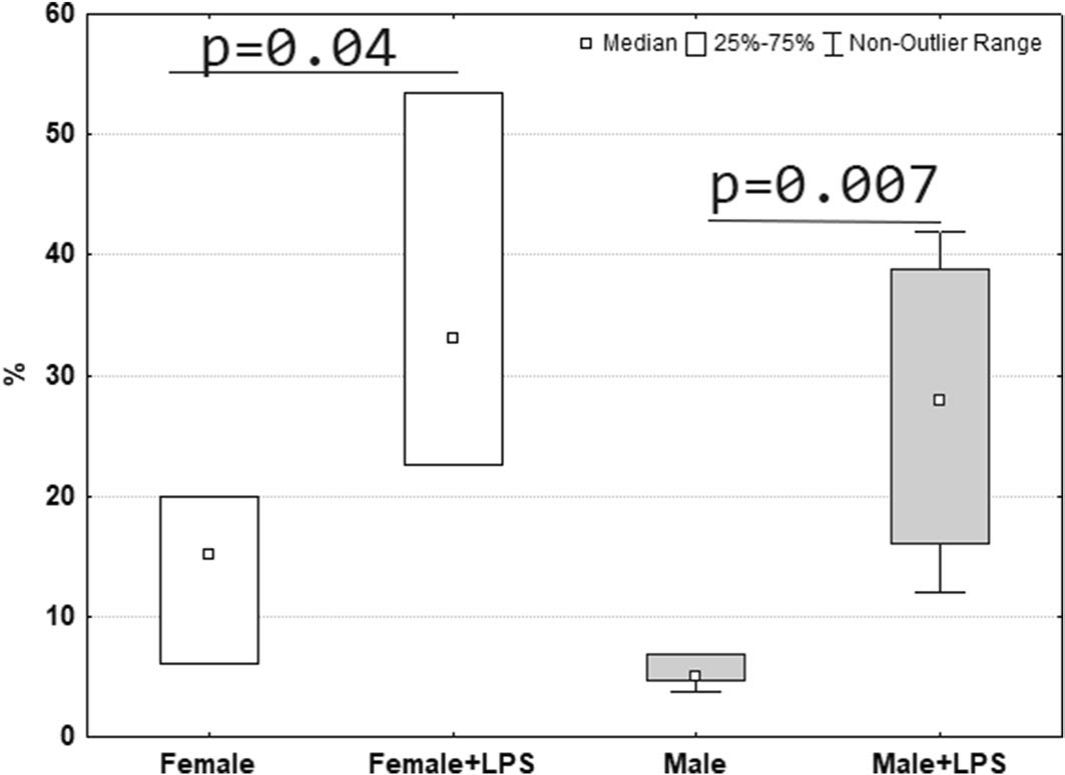
Relative number of Annexin + apoptotic dying cells in the thymus of female and male pups of Wistar rat 1 day after LPS injection.

A day after the administration of LPS, the relative content of apoptotically dying cells in the thymus of ten-day-old female and male Wistar rats increased in comparison with the corresponding control group (Fig. 5). Sex differences in the number of Annexin + thymic cells were not found (Fig. 5).

#### Peripheral blood lymphocytes subpopulations in female and male pups of Wistar rats

Males and females of the control groups had similar content of lymphocytes subpopulations, except absolute number of T-regulatory cells, that concentration was higher in females (Table 4).

**Table 4:** Absolute number of main subpopulations of lymphocytes in the peripheral blood of female and male pups of Wistar rat 1 day after LPS injection.

10^6^/ml	Groups	Sex		P^f-m^
		Female	Male	
CD3 + T cells	Control^1^ (n = 8)	4.3 (3.5–7.0)	4.4 (2.3–5.3)	0.6
	LPS^2^ (n = 10)	1.7 (1.4–1.9)	2.2 (2.0–2.5)	**0.03**
	P^1–2^	**0.008**	0.1	
CD3 + CD4 + helpers T cells	Control^1^ (n = 8)	3.4 (2.8–5.4)	3.0 (1.6–4.1)	0.5
	LPS^2^ (n = 10)	1.5 (1.0–1.7)	1.9 (1.8–2.0)	**0.04**
	P^1–2^	**0.006**	0.1	
CD3 + CD8 + cytotoxic T cells	Control^1^ (n = 8)	1.2 (1.0–2.2)	1.2 (0.8–1.7)	0.5
	LPS^2^ (n = 10)	0.4 (0.2–0.5)	0.5 (0.4–0.5)	0.07
	P^1–2^	**0.006**	**0.006**	
CD3-CD45R + B cells	Control^1^ (n = 8)	3.0 (2.2–3.6)	2.3 (1.2–3.8)	0.4
	LPS^2^ (n = 10)	1.8 (0.9–3.1)	0.3 (0.2–1.0)	0.07
	P^1–2^	0.1	**0.04**	
CD4 + CD25 + Foxp3 + regulatory T cells	Control^1^ (n = 8)	1.1 (0.95–1.1)	0.4 (0.2–0.4)	**0.005**
	LPS^2^ (n = 10)	0.07 (0.02–0.3)	0.3 (0.2–0.4)	0.2
	P^1–2^	**0.002**	0.3	

Bold - statistically significant difference, *p* < 0.05.

After the LPS injection to males and females, subpopulations of B- and T-cells, including T-helpers, T-cytotoxic lymphocytes, mostly decreased (Table 4). However significant decrease of CD3 + , CD3 + CD4 + , CD3 + CD8 + cells was revealed in females. When in males only CD3 + CD8 + lymphocytes decreased. Besides in males the absolute numbers of B-cells also significantly decreased, when in females did not change (Table 4). The number of T-regulatory cells decreased only in females. Thus, on the first day after LPS injection a decrease of T-cells in peripheral blood was more pronounced in females of pups Wistar rats, but in males the number of B-cells decreased (Table 4).

#### Cytokines production level by ConA activated spleen cells in female and male pups of Wistar rats

The level of IL-2 spleen cells production in female of control group was higher than in males (Table 5). After the administration of LPS in both female and male pups the production levels of IL-2, IL-4 and TNF-α decreased (Table 5).

**Table 5:** Cytokines level production by ConA stimulated spleen cells of female and male pups of Wistar rat 1 day after LPS injection.

Cytokine, pg/ml	Groups	Sex		P^f-m^
		Female	Male	
IL-2	Control^1^ (n = 8)	8000 (6672–8000)	3214 (1136–3619)	**0.02**
	LPS^2^ (n = 10)	2865 (636–6138)	459.7 (0–1034)	**0.03**
	P^1–2^	**0.003**	**0.02**	
IL-4	Control^1^ (n = 8)	47.2 (7.2–126.3)	15.6 (12.6–51.2)	0.07
	LPS^2^ (n = 10)	8.3 (0.5–44.5)	8.8 (3.5–14.3)	0.5
	P^1–2^	**0.03**	**0.04**	
IL-6	Control^1^ (n = 8)	1992 (1634–2976)	1925 (529–3322)	0.8
	LPS^2^ (n = 10)	2243 (1392–3492)	3173 (1680–3793)	0.9
	P^1–2^	0.8	0.5	
TNF-α	Control^1^ (n = 8)	266.8 (41.6–820.9)	246.5 (134.6–372.6)	0.4
	LPS^2^ (n = 10)	19.1 (0–85.2)	53.2 (42.9–68.2)	0.4
	P^1–2^	**0.03**	**0.02**	
IFN-γ	Control^1^ (n = 8)	85.5 (13.7–695.2)	9.8 (5.8–219.8)	0.2
	LPS^2^ (n = 10)	61.4 (7.8–211.3)	9.8 (6.8–21.9)	0.5
	P^1–2^	0.4	0.8	

Bold - statistically significant difference, *p* < 0.05.

Consequently, immune reactions in male and female pups at SIRS have similar features. We noticed the atrophy of the thymus and increase in Annexin + dying thymic cells, suppression of IL-2, IL-4 and TNF-α production by splenic cells, raising of neopterin concentration in the blood serum at both sexes. However, the level of neopterin was higher in females with SIRS than males, and only in females there was decrease in T-lymphocytes, T-helpers, T-regulatory cells, that can be reflection of immunosuppression state.

## Discussion

Immune reactions play an important role in the development of inflammatory diseases, determine their severity and outcome. An acute inflammation during SIRS and sepsis differs between males and females, with improved clinical course and increased survival rates detected in females^6,16,17^. Sex differences between individuals of reproductive age are explained by different levels of sex steroids^18^. The literature mostly provides information on sex differences in the frequency of development, severity, and mortality rates from sepsis in children during neonatal and puberty compared with adults^19^. However, there is a lack of data about sex differences in the morphofunctional state of the immune system in prepubertal period as well as the features of immune and inflammatory responses in SIRS both at prepubertal males and females. In this work, we primarily characterized the differences in the course of SIRS in 10-day-old male and female rats. It was found that despite the absence of differences in the concentration of sex steroid hormones in female and male prepubertal rats, LPS-induced inflammatory and immune reactions have some sex differences.

It is well-known that the content of sex steroid hormones in the prepubertal period is low and the concentration of estradiol and testosterone in the serum does not differ in experimental animals of different sexes, as well as in children of prepubertal period (3–6 years old)^11^. We found out absence of sex differences in estradiol (p = 0.8) and testosterone (p = 0.7) levels in 10-day-old pups, and their concentrations were low compared to the mature period^10^. Nevertheless, a day after the LPS injection an increase in the concentration of both estradiol and testosterone was observed in males, but not in females. It is possible that sex differences in hormonal changes at SIRS are due to LPS-induced damage of the hypothalamus–pituitary–adrenal (HPA) and the hypothalamus-pituitary-gonads (HPG) axis in males, which led to changes in the metabolism and synthesis of sex steroids by the testes^20^. According to the literature, an increase in estradiol concentration is an unfavorable factor in inflammatory reactions and contributes to the synthesis of LPS—binding protein (LBP)^21^. The LPS + LBP complex activates TLR4, which leads to the production of pro-inflammatory cytokines and to more pronounced inflammatory response in males^22^.

Unlike sex steroid hormones, the concentration of corticosterone in the control group differed between females and males. According to the data of R.D. Romeo et al.^23^, independently from the breed and sex, the corticosterone level in prepubertal rodents is higher than in sexually mature animals, however, the authors did not reveal sex differences in the corticosterone level in intact animals. In this work, it was demonstrated that in prepubertal female Wistar rats, the concentration of corticosterone is 6 times higher than in males. Perhaps, it is due to we have studied 10-day-old rats, while R.D. Romeo et al.^23^ have used 6 weeks old animals. However, according to study of Rolfsjord et al.^24^, salivary cortisol levels in children at 2 years old are higher than in infancy, and are higher in girls than in boys at 2 years of age. And during puberty, basal cortisol levels increase in girls, but decrease in boys as well as in response to CRH girls’ total cortisol response increases throughout the pubertal stages, whereas boys’ total response remains relatively stable across puberty^25^. Also well-known that corticosterone regulates apoptosis of thymic cells during processes of negative selection of T-lymphocytes^26^. Apoptosis of thymocytes are pronounced in prepubertal period due to the immune system undergoes the functional maturation. Therefore, the revealed in our research higher rates of Annexin + thymic cells in females of the control group may be associated with a high content of corticosterone and, probably, more active processes of maturation of the immune system.

During the prepubertal period there are maturation of not only the immune system, but hormonal and neural systems too. Exposure to LPS during this period can lead to enduring changes in brain functioning, including of HPA and HPG axis activation^22,27,28^. In the study of O. Girard-Joyal et al.^29^ was revealed that LPS treatment significantly increased serum corticosterone concentration in all mice regardless of sex and age. The authors did not reveal sex differences in the level of corticosterone in prepubertal rats in the early periods (2 h) after the injection of 1.5 mg/kg LPS, maybe because the cascade of inflammatory reactions had not yet fully developed. We used a higher dose of LPS − 15 mg/kg, evaluated changes in corticosterone after 24 h, and established a multidirectional change in the corticosterone level in pups: females had decreased level of corticosterone, but males had adaptive increasing of ones. It is possible, the LPS-induced activation of HPA axis in females was blocked by a high initial corticosterone content, which did not allow developing of an adequate stress response to LPS similar to males^30^. In rats, it was investigated a sex difference in basal corticosterone levels: females demonstrate higher corticosterone concentration than males^31–33^. Moreover, to higher hormonal reaction to stress, female rats have a delayed return to baseline ACTH and cortisol levels after acute stress, demonstrating sex differences in negative feedback regulation of the HPA axis^34–37^. Sex differences could be noticed in neural pathways that may give an answer to this sex-biased inhibition. In limbic structures known to provide inhibitory effects to the HPA axis following acute restraint stress is reduced in females in comparison to males^38^. However, during the prepubertal period the HPA axis faces active maturation. For instance, the negative feedback process is not absolutely developed in prepubertal rats. This inefficient feedback way explains particular age-related differences in HPA axis function such as the longer production of corticosterone in prepubertal rats^39^. At the same time, CRH-responsive cells in the anterior pituitary reach adult levels only on 60 days old rats, whereas they are almost all non-responsive to CRH at birth^40^.

Considering the immunomodulatory role of sex steroids, we could expect the absence of sex differences in the course of inflammatory reactions in prepubertal animals. In our study, the mortality rate from LPS in 10-day-old males was 56% (13 rats out of 23) and it was higher than in females (17%, 2 rats out of 12). Our results are consistent with literature data that revealed high mortality rates from infectious and inflammatory diseases in prepubertal boys^19^. Probably, high mortality rates in males are associated with the more pronounced inflammatory reactions in the target organs that we had identified: the area of necrosis in the liver and the number of neutrophils in the lungs were higher in males. In the literature it was shown that in the puberty, LPS causes less hypothermia and symptoms of sickness in females than in males^41^. The authors explain the revealed sex differences by the immunomodulatory effect of sex hormones^41^. However, in this work, similar results were obtained in pups with a low level of sex steroid hormones, that indicates the important role of the genes located in the sex chromosomes that take part in the regulation of LPS-induced inflammatory reactions^6,42^. One of the mechanisms, determining sex differences of inflammation in prepubertal rats, may be the activation of the X chromosome genes encoding the main proteins involved in the NF-κB dependent synthesis of pro-inflammatory mediators^14^. The mosaicism of the cells in females, determined by the inactivation of either the maternal or paternal X chromosome, provides polymorphism in the expression level of pro-inflammatory cytokines and factors which are responsible for the development of an effective immune response in infectious and inflammatory diseases^43^. In addition, it was shown that 15% of X-linked genes in females are not completely inactivated, which increases the level of production of factors and proteins involved in the development of inflammation^44^. Thus, the polymorphism of the X chromosome genes, and the mosaicism of cells in females due to inactivation of the paternal or maternal X chromosome, may be one of the causes of the sex differences in the course of inflammatory diseases and low mortality from it in females in comparison to males of prepubertal ages^6^.

Despite the higher mortality rates and the severity of inflammatory reaction in the target organs in males with SIRS, the level of endotoxin in blood serum was 10 times higher in females. Differences in endotoxin levels may be due to the distinctions in its speed metabolism and elimination by binding to LPS-binding proteins (LBP), chylomicrons, low-affinity antibodies, deacylation, dephosphorylation, acute-phase proteins^45^. Sex differences of LPS transport have not been studied yet. In the literature are most described LPS transport by LBP and lipoproteins^46^. Probably, the high level of endotoxin in the blood in females is due to differences in the concentration of LBP, which depend on the level of estrogen^21^. In prepubertal females, which not sufficiently synthesized LBP, elimination of endotoxin possibly occurs predominantly through lipoproteins^47^. The paradoxically high level of endotoxin in the blood of females with SIRS can be associated with a high contamination of the intestinal microflora with gram-negative bacteria, so it’s been shown that the number of Bacteroides in the intestinal microflora in mature females is 802 times higher than in males^48^. Besides, it was shown that myeloperoxidase of neutrophils can regulate level and inhibit activity of endotoxin^49^, so low endotoxin level in the blood of prepubertal males may be due to inhibition of myeloperoxidase of neutrophils, because of their number were higher in the male lungs.

A high level of endotoxin in females may be due to the increased permeability of the intestinal barrier to the endogenous endotoxin. Corticosterone is known to increase the permeability of the small intestine epithelial barrier. According to Zheng et al.^50^ subcutaneous injections of corticosterone lead to a decrease in the expression of genes encoding the proteins of tight contacts of the intestinal epithelium, the development of inflammatory reactions in the mucous membrane, and, ultimately, to increase the permeability of the intestinal epithelium. Thus, a high concentration of corticosterone in intact females could mediate sex differences in the permeability of the intestinal barrier for endotoxins of obligate intestinal microflora, and also, on the other hand, reduce the damaging effect of LPS on liver and lung cells.

But in comparison with females, pathological changes in the liver and inflammation in the lung as well as mortality rates were higher in males with lower endotoxin levels. Probably, less pronounced alterative and inflammatory changes in the target organs in females against the background of severe endotoxemia are associated with the high content of the anti-inflammatory corticosterone in the blood serum. It was shown that cortisol reduces the degradation of IκBα and phosphorylation of IκB α in a dose-dependent manner, so as inhibits MAPK phosphorylation in LPS-induced RAW264.7 cells, demonstrating a significant inhibitory effect on NF-κB activity and as result decrease in production of PGE2, IL1β by macrophages of RAW 264.7 cell line^51^.

LPS, which is derived from the cell wall of Gram-negative bacteria, is commonly used as an immune challenge in laboratory rodents^52^. It binds to TLR-4 and activates an intracellular signaling pathway via NF-kB^53^ resulting in the transcription and translation of immune regulated proteins called cytokines^53,54^. In response to the LPS injection in both prepubertal females and males, a disturbance of the balance between pro- and anti-inflammatory response was observed. That was characterized by a decrease in the production of pro- and anti-inflammatory cytokines (IL-2, IL-4, TNF-α, TGFβ), the number of CD3 + T-cells, CD3 + CD8 + lymphocytes, atrophy of the thymus with apoptotic death of cortex lymphocytes. According to the literature, the immune response in prepubertal mice is not expressed during abdominal sepsis, which is apparently associated with reduced expression of genes (*Mhc II, Cd40, Btla il1β, Arg2*), responsible for the proliferation of immune cells, differentiation of T- lymphocytes, development and maturation of blood cells and white blood cells^55^. Apparently, due to the functional immaturity of the immune system in prepubertal animals of both sexes, adaptive activation of the immune response does not occur after the injection of LPS, and the immune reactions shift towards CARS (compensatory anti-inflammatory response syndrome).

The severity of SIRS and the development of immunosuppression in prepubertal females and males is evidenced by a decrease in CD4 + CD8 + lymphocytes. The severity of experimental abdominal sepsis is accompanied with the decrease in the number of cytotoxic T-lymphocytes^56^. In clinical studies the decrease of cytotoxic T-lymphocytes in septic patients is considered as the evidence of immunosuppression^57^.

Severe apoptosis of thymic cells in prepubertal females and males is also an unfavorable factor in the development and course of SIRS. One of the possible mechanisms mediating activation of apoptosis of thymus cells is the increase in serum glucocorticoid levels^58^, receptors for which are expressed on the surface of thymocytes^59^. Double positive lymphocytes (CD4 + CD8 +) are most sensitive to glucocorticoids, that activated in thymic cells a caspase-dependent intracellular cascade, leading to their apoptosis^60^. Mature T-lymphocytes (CD4 + or CD8 +) are more resistant to glucocorticoids, due to the activation of the CD28-dependent intracellular cascade, which inhibits the transcription of apoptosis genes Bcl-X (L)^26^. In addition to corticosterone-dependent apoptosis of thymocytes, an important role in this process is played by pro-inflammatory cytokines (TNF-α and IFN-γ), whose production level increases with SIRS, infectious and inflammatory diseases^61^. LPS also has a direct negative effect on thymic cells by activating TLR4 receptors on thymocytes^62^. It is possible that both mechanisms are involved in the induction of apoptosis of thymus cells in females and males: in males, an increase in the concentration of corticosterone plays a large role in the mechanisms of thymocyte death, and in females—endotoxin.

However, we also identified sex differences in immune responses in SIRS. In comparison to males, ex vivo production of IL-2 was higher in females of both control group and with LPS. One of the functions of IL-2 is to maintain the differentiation and maturation of regulatory T cells in normal conditions^63^. A high level of IL-2 production in females of the control group was combined with a large number of T-regulatory (Treg) cells in the blood, while in males, IL-2 production and Treg content were lower than in females. The content of other studied lymphocyte subpopulations and the production of cytokines in females and males of this age did not differ. According to the literature in the reproductive period in humans^64^ and experimental animals^10,65^, the number of CD3 + CD4 + lymphocytes is higher in females, and CD3 + CD8 + – in males, which is most likely determined by differences in the content of sex steroid hormones.

In comparison to males, in females with SIRS the absolute number of CD3 + CD4 + T-helpers and CD4 + CD25 + Foxp3 + Treg cells in the blood decreased, while the content of CD3-CD45R + B-lymphocytes did not differ from the control group. In prepubertal females with SIRS, the concentration of neopterin, a marker of the activation of cellular immunity, did not change. That’s all reflect the predominant activation of humoral immune reactions. A decrease in CD3 + CD4 + lymphocytes in the peripheral blood in the early stages of the disease in patients with sepsis is noted both in reproductive age and in children aged 1 to 18 years, and the number of T-lymphocytes negatively correlates with the severity of sepsis^66–70^. In experimental models of sepsis at the early stages of its development, a decrease in the number of Treg in the blood is combined with high mortality rates^71^.

In contrast to females, we observed a decrease in the absolute number of B-cells in males with SIRS. It was combined with an increase in the concentration of neopterin in the blood serum and the absence of a changes in the number of T-helpers and Treg. Apparently, in males, the acute LPS-induced inflammatory reaction occurs with the predominant activation of cellular immunity reactions. Pronounced pathological changes in the target organs found in prepubertal males with SIRS may be the result of a polarization of the cellular immune reactions^72^.

**Figure 6:**
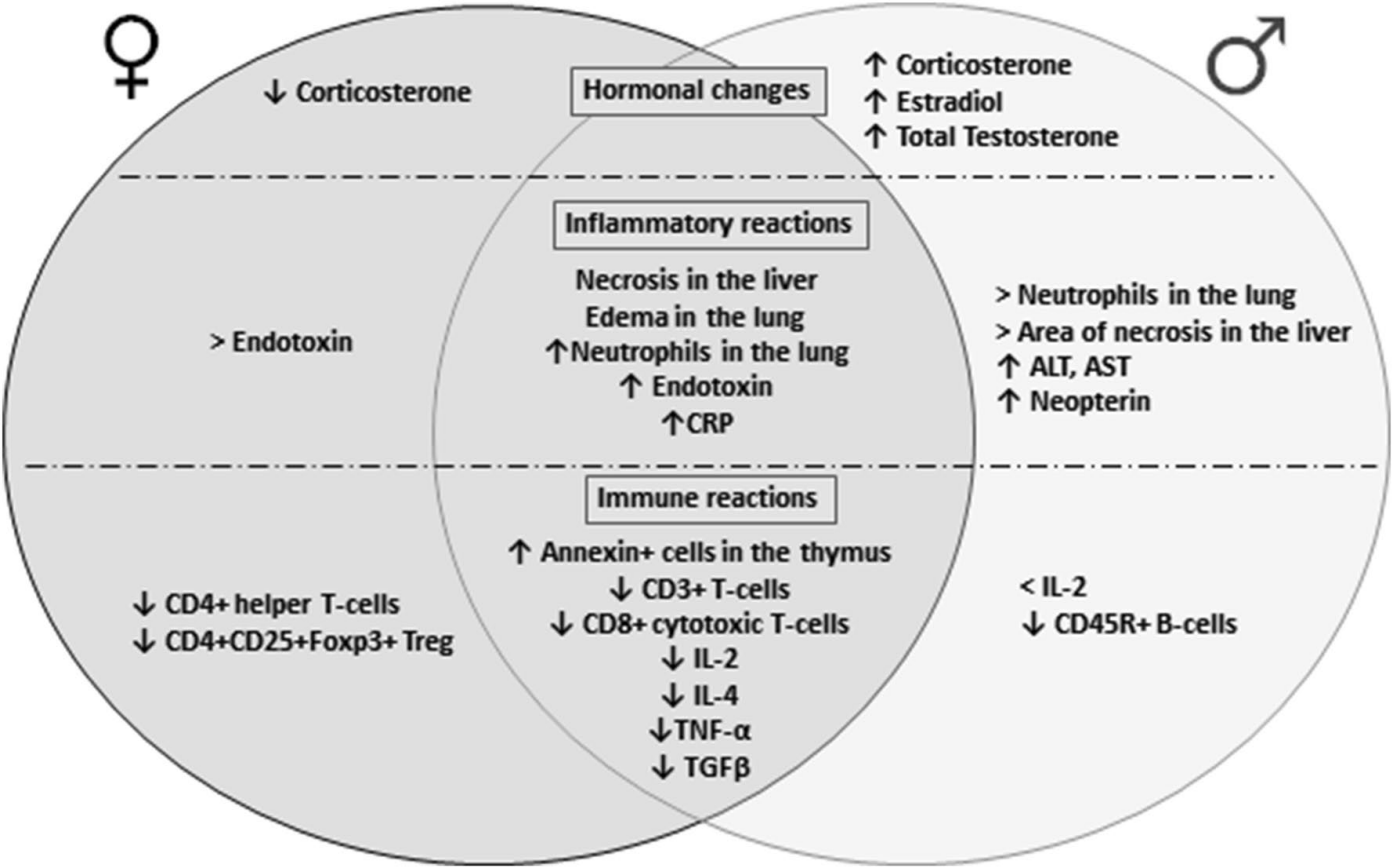
Sex differences of LPS-induced changes in hormonal levels, immune and inflammatory reactions in prepubertal rats. Hormonal changes: In contrast to females in males with SIRS, there is an increase in the concentration of hormones—corticosterone, estradiol and total testosterone. And in females there is only a decrease in the corticosterone content. Inflammatory changes: there are SIRS symptoms in both males and females with SIRS—inflammatory changes in target organs, endotoxemia, and increased endotoxin levels. But there are sex differences—the morphological manifestations of SIRS were more pronounced in males. They have higher number of neutrophils in the lung, area of necrosis in the liver, activity of ALT and AST and neopterin content. Nevertheless, the endotoxin level is higher in females with SIRS than in males. Immune reactions: in males and females, there are a suppression of the production of pro- and anti-inflammatory cytokines (IL-2, IL-4, TNF-α, TGF-β) and a decrease in the number of CD3 + cells, including CD3 + CD8 + cytotoxic T-lymphocytes. However, the peculiarities of immune reactions are revealed in animals of different sexes after the LPS injection: IL-2 production in males is lower than in females, and the number of CD45R + B-lymphocytes in the male blood significantly decreases, while in females the number of CD3 + CD4 + T-helpers and CD4 + CD25 + Foxp3 + Treg cells decreases.

Thus, we showed first that the course of the inflammatory response in the liver and lungs is more pronounced in males, while immunosuppressive reactions, such as thymic atrophy, decrease in production pro- and anti-inflammatory cytokines, the number of T-lymphocytes, in particular, cytotoxic T-lymphocytes, in prepubertal animals of both sexes are equally expressed. Moreover, in animals with SIRS in the prepubertal period, sex differences in the immune response are observed: suppression of the cellular immune response was observed in females, and on the contrary, suppression of the adaptive immunity was observed in males (Fig. 6).

## Conclusions

To sum up, despite the absence of sex differences in estradiol and testosterone levels, LPS-induced inflammatory changes in liver and lungs are more pronounced among males. On the contrary to inflammatory changes, the LPS-induced immune reactions in females and males are similar and are characterized by immunosuppression. Apparently, sex differences of inflammatory reactions depend on X/Y chromosomes gene expression, but not sex hormones. However, the least expressed sex differences in immune responses in the prepubertal period can be determined by the low levels of sex steroids and the absence of their immunomodulatory effect.

Also in males, endotoxin level after LPS injection is tenfold lower than in females. Thus, endotoxin cannot be used as a marker of the severity of multiple organ failure in prepubertal period.

Thus, there is a lack of literature data about animals and humans’ immune system sex differences during the prepubertal period. In our work we firstly revealed existence of sex differences of pups’ inflammatory reactions. Further studies require the identification of mechanisms, determining the sex differences in the inflammatory and immune responses in prepubertal animals in order to develop more effective immunotherapy and minimize side effects of children vaccination.

## Methods

### Experimental animals

In the study 10 days old female (n = 21) and male (n = 30) pups of Wistar rats and weighting 20–25 g were used. To obtain 10 days old pups we used 15 mature Wistar rats (10 females and 5 males). The durations of pregnancy in females were 21–23 days. The offspring of one female was represented by 6–12 individuals. Male and female Wistar rats, 2–3 months old and weighing 200–250 g, were purchased from the animal breeding facility of the federal budget institution for science, the “Scientific Centre for Biomedical Technology of the Federal Medical and Biological Agency”. The study received permission from the Bioethics Committee of the Science Research Institute of Human Morphology (Protocol No. 18a, December, 22, 2016). All manipulations with animals were carried out according to the European convention for the protection of vertebrate animals used for experimental and other scientific purposes (ets no. 123), Strasbourg, 2006. Three rats (2 females and 1 male) per cages (18.5 × 60x38 cm) were housed in temperature-regulated room at 12:12 h light–dark cycle, relative humidity, between 55 and 65%; and unlimited access to water and food (“Char”, JSC “Range-Agro”, Russia).

### Modelling of systemic inflammatory response syndrome (SIRS)

Male and female pups in the experimental groups were injected intraperitoneally with dissolved in 9% NaCl lipopolysaccharide (LPS) from E. coli O26:B6 (Sigma, USA) in dose of 15 mg/kg, which is a model of endotoxin shock and sublethal for adults mature rats^10^. The pups in the control groups received an intraperitoneal injection of physiological saline. Both control groups of male and female rats consisted of 8 rats, the experimental group of females included 12 rats, and of males—23. A higher quantity of animals in the groups, especially males, receiving high-dose LPS is explained by the high rates of mortality. Some animals were dying during 6 h after LPS injection. Mortality rates of pups in response to the injection of LPS were 2 out of 12 (17%) in females and 13 out of 23 (56%) in males.

### Sample collection

We used Zoletil at a dose of 15 mg/kg body weight for anaesthesia (Virbac Sante Animale, France). The animals were euthanized after 24 h of LPS injection. Venous blood from jugular veins^73^ was centrifuged for 20 min at 200 g. The obtained serum was frozen at -70ºC and stored for no more than 2 months. The thymus, liver and lungs were fixed in Bouin's solution (75 ml picric acid, 25 ml formalin, 5 ml glacial acetic acid)^74^ for 24 h, and organs were embedded in paraffin according to routine procedures. Histological sections of 4–5 µm thick were produced and stained with haematoxylin and eosin (“BioVitrum”, Russia).

### Morphological study

The histological slides were randomized and blinded. Using the light microscopic method, the number of neutrophils in the intra-alveolar septae of the lungs was counted in 10 high-power fields (25,000 μm^2^) per section, and the average number of neutrophils per slide was determined.

The area of necrosis in the liver was estimated using the Image Scope M program interactively using a Leica DFC290 camera. The measurements were carried out on the entire area of histological slice, the results were showed in μm^2^.

In the histological slices of the thymus, the volume fraction of the functional zones of immune organ was determined by the point-count method.

### Biochemical analysis

To estimate the severity of liver damage, the activity of the indicator enzymes aspartate aminotransferase (AST) and alanine aminotransferase (ALT) in rat serum was determined (DiaSys, Germany) using a blood biochemistry semi-automatic analyser (Clima MC-15; RAL, Spain).

### Isolation and cultivation of splenic cells

Isolation and cultivation of splenic cells were carried out as describe in^10^. For isolation of splenic cells, a piece of spleen was aseptically remove from each rat, placed in Potter homogenizer containing the Roswell Park Memorial Institute (RPMI) 1,640 medium and single-cell suspensions were prepared. The red blood cells were lysed by distilled water. To activate cytokine synthesis and secretion, we cultivated 10^6^/ml spleen cells in 1 ml of culture medium with concanavalin A (5 µg/ml) for 20 h at 37 °C and 5% CO_2_ in 24-well cultured plates. The culture medium consisted of RPMI-1640 (PanEco, Russia), 5% inactivated foetal bovine serum (FBS), 2 mM glutamine and 50 µg /ml gentamicin^75^. The cell viability was determined according to trypan blue exclusion^6^.

### ELISA

We estimated the concentration of corticosterone (IBL, Germany), total and free testosterone (DBC, Canada), estradiol (DBC, Canada), neopterine (IBL, Germany), C-reactive protein—CRP (Clone-Cloud Corp., China) and TGF-β (eBioscience, USA) in the serum by ELISA. The endotoxin level in the serum was estimated by chromogenic LAL test (HBT, USA). In the culture fluid of splenic cells, we measured the concentrations of IL-2, IL-4, IL-6, TNF-α, and IFN-γ by ELISA test systems from eBioscience (USA).

### Apoptosis of thymic cells

As it had been described previously^6^, to determine the number of apoptotic cells in the thymus, a suspension of cells at a concentration of 10^6^/ml was stained with antibodies (anti-rat CD3 as marker of T-lymphocytes) conjugated to PE (phycoerythrin). Then, the stained cell suspension was incubated with annexin V (Annexin V FITC Kit, Beckman Coulter, USA) conjugated to FITC (fluorescein isothiocyanate) and with propidium iodide (PI). Flow cytometry evaluation of apoptotic cells (Annexin + PI-) was performed on a Cytomics FC 500 (Beckman Coulter, USA).

### Flow Cytometry

Absolute and relative numbers of lymphocytes of T- and B-cells subpopulations in peripheral blood were counted using flow cytometry (Beckman Coulter, USA). The following antibodies (eBioscience) were used for immune phenotypic analysis of the main subpopulations of lymphocytes: anti-rat CD3 (CD3^+^ T-lymphocytes); anti-rat CD4 (CD3^+^CD4^+^ T-helpers); anti-rat CD8a (CD3^+^CD8^+^ cytotoxic T cells); anti-rat CD45R (CD3^-^CD45R^+^ B-lymphocytes); anti-rat CD25 and anti-mouse/rat Foxp3 (CD4^+^CD25^+^Foxp3^+^ regulatory T cells). Erythrocytes were lysed with OptiLyse C solution (“eBioscience”, USA).

### Statistical analysis

Digital data were tested for normality using the Kolmogorov–Smirnov test in Statistica 8.0. In case if data were distributed not normally, a nonparametric test were used to establish the reliability of the differences between the indicators: Kruskal–Wallis’s method for multiple comparison. A comparison of the normally distributed data was made using one-way analysis of variance (ANOVA) on ranks. The median and interquartile range (Me; Low–High) were calculated for values of the measured parameters. The differences were considered statistically significant when p < 0.05. At least 8 observations were presented in each group. Data are represented graphically using box-and-whisker plots, which demonstrate the median, interquartile range, lower extreme (25%), and upper extreme (75%) of the data.

## References

[1] Giefing-Kröll C, Berger P, Lepperdinger G, Grubeck-Loebenstein B (2015). How sex and age affect immune responses, susceptibility to infections, and response to vaccination. Aging Cell.

[2] Koch MA (2018). Sex bias in sepsis. Cell Host Microbe..

[3] Muenchhoff M, Goulder PJ (2014). Sex differences in pediatric infectious diseases. J Infect Dis..

[4] Vázquez-Martínez ER, García-Gómez E, Camacho-Arroyo I, González-Pedrajo B (2018). Sexual dimorphism in bacterial infections. Biol. Sex. Differ..

[5] Klein SL, Flanagan KL (2016). Sex differences in immune responses. Nat Rev Immunol..

[6] Lefèvre N (2017). Sex differences in inflammatory response and acid-base balance in prepubertal children with severe sepsis. Shock..

[7] Ghazeeri G, Abdullah L, Abbas O (2011). Immunological differences in women compared with men: overview and contributing factors. Am. J. Reprod. Immunol..

[8] Dibbern J, Eggers L, Schneider BE (2017). Sex differences in the C57BL/6 model of Mycobacterium tuberculosis infection. Sci. Rep..

[9] Matter ML (2017). High mortality due to sepsis in Native Hawaiians and African Americans: the multiethnic cohort. PLoS ONE.

[10] Kosyreva AM (2018). Sex differences of inflammation in target organs, induced intraperitoneal injection of lipopolysaccharide, depend on its dose. J. Inflamm. Res..

[11] Flanagan KL, Fink AL, Plebanski M, Klein SL (2017). Sex and gender differences in the outcomes of vaccination over the life course. Annu. Rev. Cell Dev. Biol..

[12] Casimir GJ, Mulier S, Hanssens L, Zylberberg K, Duchateau J (2010). Gender differences in inflammatory markers in children. Shock..

[13] Bindl L (2003). Gender-based differences in children with sepsis and ARDS: the ESPNIC ARDS Database Group. Intensive Care Med..

[14] Spolarics Z (2007). The X-files of inflammation: cellular mosaicism of X-linked polymorphic genes and the female advantage in the host response to injury and infection. Shock..

[15] Ghuman AK, Newth CJ, Khemani RG (2013). Impact of gender on sepsis mortality and severity of illness for prepubertal and postpubertal children. J. Pediatr..

[16] Barrow RE, Herndon DN (1990). Incidence of mortality in boys and girls after severe thermal burns. Surg. Gynecol. Obstet..

[17] Tasker RC (2000). Gender differences and critical medical illness. Acta Paediatr..

[18] Gubbels Bupp MR, Potluri T, Fink AL, Klein SL (2018). The confluence of sex hormones and aging on immunity. Front. Immunol..

[19] Ahmed R, Oldstone MB, Palese P (2007). Protective immunity and susceptibility to infectious diseases: lessons from the 1918 influenza pandemic. Nat. Immunol..

[20] Sharma R (2019). Programming effects of pubertal lipopolysaccharide treatment in male and female CD-1 mice. J. Immunol..

[21] Rettew JA, Huet YM, Marriott I (2009). Estrogens augment cell surface TLR4 expression on murine macrophages and regulate sepsis susceptibility in vivo. Endocrinology.

[22] Dunzendorfer S, Lee HK, Soldau K, Tobias PS (2004). Toll-like receptor 4 functions intracellularly in human coronary artery endothelial cells: roles of LBP and sCD14 in mediating LPS responses. FASEB J..

[23] Romeo RD, Kaplowitz ET, Ho A, Franco D (2013). The influence of puberty on stress reactivity and forebrain glucocorticoid receptor levels in inbred and outbred strains of male and female mice. Psychoneuroendocrinology..

[24] Rolfsjord LB (2017). Morning salivary cortisol in young children: reference values and the effects of age, sex, and acute bronchiolitis. J. Pediatr..

[25] Stroud LR, Papandonatos GD, Williamson DE, Dahl RE (2011). Sex differences in cortisol response to corticotropin releasing hormone challenge over puberty: pittsburgh pediatric neurobehavioral studies. Psychoneuroendocrinology..

[26] van den Brandt J, Wang D, Reichardt HM (2004). Resistance of single-positive thymocytes to glucocorticoid-induced apoptosis is mediated by CD28 signaling. Mol. Endocrinol..

[27] Sisk CL, Zehr JL (2005). Pubertal hormones organize the adolescent brain and behavior. Front. Neuroendocrinol..

[28] Brydges NM, Best C, Thomas KL (2020). Female HPA axis displays heightened sensitivity to pre-pubertal stress. Stress..

[29] Girard-Joyal O (2015). Age and sex differences in C-Fos expression and serum corticosterone concentration following LPS treatment. Neuroscience.

[30] Lenczowski MJP, Van Dam AM, Poole S, Larrick JW, Tilders FJH (1997). Role of circulating endotoxin and interleukin-6 in the ACTH and corticosterone response to intraperitoneal LPS. Am. J. Physiol. Regul. Integr. Compar. Physiol..

[31] Aloisi AM, Steenbergen HL, van de Poll NE, Farabollini F (1994). Sex-dependent effects of restraint on nociception and pituitary-adrenal hormones in the rat. Physiol. Behav..

[32] Aoki M, Shimozuru M, Kikusui T, Takeuchi Y, Mori Y (2010). Sex differences in behavioral and corticosterone responses to mild stressors in ICR mice are altered by ovariectomy in peripubertal period. Zool. Sci..

[33] Weintraub A, Singaravelu J, Bhatnagar S (2010). Enduring and sex-specific effects of adolescent social isolation in rats on adult stress reactivity. Brain Res..

[34] Viau V, Bingham B, Davis J, Lee P, Wong M (2005). Gender and puberty interact on the stress-induced activation of parvocellular neurosecretory neurons and corticotropin-releasing hormone messenger ribonucleic acid expression in the rat. Endocrinology.

[35] Handa RJ, Burgess LH, Kerr JE, O’Keefe JA (1994). Gonadal steroid hormone receptors and sex differences in the hypothalamo-pituitary-adrenal axis. Horm. Behav..

[36] Babb JA, Masini CV, Day HE, Campeau S (2013). Sex differences in activated corticotropin-releasing factor neurons within stress-related neurocircuitry and hypothalamic-pituitary-adrenocortical axis hormones following restraint in rats. Neuroscience.

[37] Iwasaki-Sekino A, Mano-Otagiri A, Ohata H, Yamauchi N, Shibasaki T (2009). Gender differences in corticotropin and corticosterone secretion and corticotropin-releasing factor mRNA expression in the paraventricular nucleus of the hypothalamus and the central nucleus of the amygdala in response to footshock stress or psychological. Psychoneuroendocrinology..

[38] Figueiredo HF, Dolgas CM, Herman JP (2002). Stress activation of cortex and hippocampus is modulated by sex and stage of estrus. Endocrinology.

[39] McCormick CM, Mathews IZ (2007). HPA function in adolescence: role of sex hormones in its regulation and the enduring consequences of exposure to stressors. Pharmacology Biochem. Behav..

[40] Senovilla L, García-Sancho J, Villalobos C (2005). Changes in expression of hypothalamic releasing hormone receptors in individual rat anterior pituitary cells during maturation, puberty and senescence. Endocrinology.

[41] Kane L, Ismail N (2017). Puberty as a vulnerable period to the effects of immune challenges: focus on sex differences. Behav. Brain Res..

[42] Balsara SL, Faerber JA, Spinner NB, Feudtner C (2013). Pediatric mortality in males versus females in the United States, 1999–2008. Pediatrics.

[43] Migeon BR (2006). The role of X inactivation and cellular mosaicism in Women's health and sex-specific diseases. JAMA.

[44] Carrel L, Willard HF (2005). X-inactivation profile reveals extensive variability in X-linked gene expression in females. Nature.

[45] Buttenschoen K, Radermacher P, Bracht H (2010). Endotoxin elimination in sepsis: physiology and therapeutic application. Langenbeck’s Arch. Surg..

[46] Wendel M, Paul R, Heller AR (2007). Lipoproteins in inflammation and sepsis II. Clinical aspects. Intensive Care Med..

[47] Vargas-Caraveo A (2017). Lipopolysaccharide enters the rat brain by a lipoprotein-mediated transport mechanism in physiological conditions. Sci Rep..

[48] Org E (2016). Sex differences and hormonal effects on gut microbiota composition in mice. Gut Microbes..

[49] Allen RC, Henery ML, Allen JC, Hawks RJ, Stephens JT (2019). Myeloperoxidase and eosinophil peroxidase inhibit endotoxin activity and increase mouse survival in a lipopolysaccharide lethal dose 90% model. J. Immunol. Res..

[50] Zheng G (2013). Corticosterone mediates stress-related increased intestinal permeability in a region-specific manner. Neurogastroenterol Motil..

[51] Dong J (2018). Cortisol modulates inflammatory responses in LPS-stimulated RAW264.7 cells via the NF-κB and MAPK pathways. BMC Vet Res..

[52] Kentner AC, Pittman QJ (2010). Minireview: early-life programming by inflammation of the neuroendocrine system. Endocrinology.

[53] Vitkovic L (2000). Cytokine signals propagate through the brain. Mol. Psychiatry..

[54] Andreakos E (2004). Distinct pathways of LPS- induced NF-kappa B activation and cytokine production in human myeloid and nonmyeloid cells defined by selective utilization of MyD88 and Mal/TIRAP. Blood.

[55] Gentile LF (2014). Protective immunity and defects in the neonatal and elderly immune response to sepsis. J. Immunol..

[56] Condotta SA, Rai D, James BR, Griffith TS, Badovinac VP (2013). Sustained and incomplete recovery of naive CD8+ T cell precursors after sepsis contributes to impaired CD8+ T cell responses to infection. J. Immunol..

[57] Boomer JS (2011). Immunosuppression in patients who die of sepsis and multiple organ failure. JAMA.

[58] Savino W (2006). The thymus is a common target organ in infectious diseases. PLoS Pathog..

[59] Herold MJ, McPherson KG, Reichardt HM (2006). Glucocorticoids in T cell apoptosis and function. Cell Mol. Life Sci..

[60] Wang D, Müller N, McPherson KG, Reichardt HM (2006). Glucocorticoids engage different signal transduction pathways to induce apoptosis in thymocytes and mature T cells. J. Immunol..

[61] Pozzesi N (2014). Role of caspase-8 in thymus function. Cell Death Differ..

[62] Tsuji T, Asano Y, Handa T (2000). Induction of apoptosis in lymphoid tissues of mice after intramuscular injection of enterotoxigenic Escherichia coli enterotoxin. Immunobiology.

[63] Arenas-Ramirez N, Woytschak J, Boyman O (2015). Interleukin-2: biology, design and application. Trends Immunol..

[64] Shahal-Zimra Y (2016). Lymphocyte subset reference ranges in healthy Israeli adults. Isr. Med. Assoc. J..

[65] Gao Y, Postovalova EA, Makarova OV, Dobrynina MT, Mikhailova LP (2018). Sex-related differences in the morphology and subpopulation composition of colon lymphocytes in experimental acute colitis. Bull. Exp. Biol. Med..

[66] Gouel-Chéron A, Venet F, Allaouchiche B, Monneret G (2012). CD4+ T-lymphocyte alterations in trauma patients. Crit. Care..

[67] Hoser GA, Skirecki T, Złotorowicz M, Zielińska-Borkowska U, Kawiak J (2012). Absolute counts of peripheral blood leukocyte subpopulations in intraabdominal sepsis and pneumonia-derived sepsis: a pilot study. Folia Histochem. Cytobiol..

[68] Wu H-P (2013). Associations of T helper 1, 2, 17 and regulatory T lymphocytes with mortality in severe sepsis. Inflamm Res..

[69] Cabrera-Perez J, Condotta SA, Badovinac VP, Griffith TS (2014). Impact of sepsis on CD4 T cell immunity. J. Leukoc. Biol..

[70] Muszynski JA (2014). Early adaptive immune suppression in children with septic shock: a prospective observational study. Crit. Care..

[71] Tatura R (2015). Relevance of Foxp3^+^ regulatory T cells for early and late phases of murine sepsis. Immunology.

[72] Kelly-Scumpia KM (2011). B cells enhance early innate immune responses during bacterial sepsis. J. Exp. Med..

[73] Parasuraman S, Raveendran R, Kesavan R (2010). Blood sample collection in small laboratory animals. J. Pharmacol. Pharmacother..

[74] Wilcox MH (1994). Comparison of formalin and Bouin's reagent for fixation of coagulase negative staphylococcal biofilm. J Clin Pathol..

[75] Lin K-H (2016). Astaxanthin, a carotenoid, stimulates immune responses by enhancing IFN-γ and IL-2 secretion in primary cultured lymphocytes in vitro and ex vivo. Int. J. Mol Sci..

